# Iodine biofortification of bean (*Phaseolus vulgaris* L.) and pea (*Pisum sativum* L.) plants cultivated in three different soils

**DOI:** 10.1371/journal.pone.0275589

**Published:** 2022-10-04

**Authors:** Viktória Vetési, Gyula Záray, Anett Endrédi, Sirat Sandil, Márk Rékási, Tünde Takács, Péter Dobosy

**Affiliations:** 1 Doctoral School of Environmental Sciences, Eötvös Loránd University, Budapest, Hungary; 2 Institute of Aquatic Ecology, Centre for Ecological Research, Budapest, Hungary; 3 Institute for Soil Sciences, Centre for Agricultural Research, Budapest, Hungary; United Arab Emirates University, UNITED ARAB EMIRATES

## Abstract

An important challenge for mankind today is to find a plant-based source of iodine, instead of table salt, which would provide the recommended daily dosage of iodine. The aim of this work was to study the accumulation of iodine and the physiochemical changes in bean (*Phaseolus vulgaris* L.) and pea (*Pisum sativum* L.) irrigated with iodine-containing water. Applying iodine at concentration of 0.5 mg L^-1^ resulted 51, 18, and 35% decrement in biomass of bean fruit, while in pea fruit, a 13% reduction and a 3 and 2% increment were observed when the plants were cultivated in sand, sandy silt, and silt, respectively. The highest iodine concentrations in the bean and pea fruits were detected in plants cultivated in silt soil with concentration of 0.5 mg I^-^ L^-1^ and amounted to 1.6 and 0.4 mg kg^-1^, respectively. In presence of iodine at concentration of 0.5 mg L^-1^, the concentration of magnesium, phosphorous, manganese and iron increased in the bean fruit, while in the case of pea, at iodine concentration above 0.1 mg L^-1^ the uptake of these nutrients were hampered. Based on these facts, the iodized bean can be recommended as a possible food source to enhance the iodine intake.

## Introduction

Iodine (I), as a vital compound of thyroxine (3,5,3’,5’-tetraiodothyronine) and triiodothyronine (3,5,3’-triiodothyronine) hormones, is essential for humans. These hormones regulate several metabolic processes in the human body, including fetal and early growth, protein synthesis, as well as the functioning of the nervous system and brain [1,2]. Both insufficient and excessive iodine intake can affect the synthesis of thyroid hormones. Higher iodine consumption (more than the maximum amount of 1100 μg/day; the maximum tolerable value of 600 μg/day set by the Scientific Committee on Food) leads to thyrotoxicosis and autoimmune thyroid problems, while iodine deficiency results in hypothyroidism, goitre, brain damage, and other diseases such as cretinism and stomach cancer [3–6]. Iodine deficiency disorders (IDDs) affect ca. 2.2 billion people around the globe [4,7]. Based on the recommendations of the World Health Organization (WHO), United Nations Children’s Fund, and the International Council for Control of Iodine Deficiency Disorders, the dietary allowance for the age groups 0–5 years, 6–12 years, and >12 years is 90, 120, and 150 μg/day, respectively [3,5,8].

Similar to other essential elements (e.g., zinc, iron, selenium), iodine also has to be taken up from external sources, and 90% of iodine is supplied by food (e.g., seafood, bread, dairy products, macroalgae, etc.) and 10% by water [2]. However, the iodine content of these available foodstuffs is not enough to supply the recommended intake [8,9]. The most commonly used strategy for IDDs is salt iodination, but, i) during cooking, the iodine content in processed foods can be reduced by more than 20% [10]; and ii) excessive salt intake can cause high blood pressure and other cardiovascular diseases, therefore, the WHO proposed a 30% reduction in salt consumption by 2025 [11].

Iodine is a non-essential micronutrient for plants, but they can accumulate iodine from the soil and soil solution where iodine occurs both in inorganic (iodide, iodate) and organic forms. The iodine concentration of soils ranges between <0.1 and 150 mg kg^-1^ and its chemical form and mobility depend on the soil composition e.g. texture, pH, redox conditions, and anion exchange capacity [12–15]. In many areas (e.g., Europe, Africa, Southeast Asia), high rainfalls, floods, erosion, and overgrazing can significantly reduce the iodine content of the soil. Due to these phenomena, plants grown in these soils have low iodine content in their edible parts, and thus, do not contain sufficient iodine to cover the daily iodine requirement of the human body [12,13,16].

Agronomic biofortification is a promising alternative for providing iodine-enriched plants by using fertilizer, hydroponic, or irrigation technologies [17,18]. Several experiments have focused on increasing the iodine concentration in edible parts of plants such as green bean [19]; garden pea [20]; cabbage [21]; tomato [21–27]; carrot [26,28–30]; potato [28,31]; cowpea [32], and lettuce [19,26,33].

Based on the literature data listed above, phytotoxicity symptoms have been reported at potassium iodide concentrations of more than 1660 mg L^-1^ in tomato plants (soil and hydroponic experiments) [23,24], 80 mg kg^-1^ in potato plants (soil treatment) [31] and 10–15 kg ha^-1^ in cowpea plants (spraying treatment) [32]. However, in other studies, toxic effects in potato [28], carrot [28–30], and tomato plants [21,22] were not observed. A decrease in biomass has been reported at iodine concentrations of more than 50 mg kg^-1^ in tomato (soil treatment) [26], 80 mg kg^-1^ in potato (soil treatment) [31], 5 mg L^-1^ in lettuce (hydroponic system) [33], and 0.5 mg L^-1^ in lettuce (through irrigation water) [19] plants. Applying iodine treatment in relatively low concentrations (0.6 mg L^-1^ and 1.0 mg kg^-1^) resulted in stimulation on the biomass production of tomato plants [22,29], significant increment in phosphorous content and a decrement in iron concentration were observed, however, concentrations of magnesium, copper, manganese and zinc in carrot plants were not influenced [30]. In other studies, using potassium iodide-containing irrigation water resulted in a reduced concentration of iron and magnesium in the edible parts of cabbage, tomato [21], bean, lettuce [19], carrot, and potato plants [28].

To the best of our knowledge based on the available literature, the biofortification of plants with iodine by using iodine-containing irrigation water has not been investigated. Furthermore, any of the published works have not studied the growth of the test plants in different soil types with varying physio-chemical properties. In this paper, iodine accumulation in green bean (*Phaseolus vulgaris* L., cv. ‘Golden Goal’) and garden pea (*Pisum sativum* L., cv. ‘Rajnai törpe’) grown in sand, sandy silt, and silt soil was investigated by irrigating the plants with different concentrations of iodine-containing water. The changes in photosynthetic efficiency, dry mass, and concentration of iodine as well as selected macro-and microelements were followed. According to our hypothesis, the usage of iodine-containing water for irrigation increases the concentration of iodine in the different plant parts. It can be also hypothesized that the chemical composition of the different cultivation mediums may influence biomass production and the transport of iodine and essential elements to the edible plant parts.

## Materials and methods

### Chemicals

All chemicals applied in the experiments were of analytical grade. For dilution and preparation of standard solutions, ultra-pure water (18 MΩ cm^-1^) was produced using WasserLab Autwomatic equipment (Labsystem Ltd., Budapest, Hungary). The standard solution was prepared from solid potassium iodate, and a multi-element standard solution (Sigma Aldrich Ltd., Hungary) was used for the quantitative determination of iodine, boron (B), magnesium (Mg), phoshorous (P), manganese (Mn), iron (Fe), copper (Cu) and zinc (Zn). The accuracy of the analytical measurements was verified using NIST SRM 1573a tomato leaf certified reference material (National Institute of Standards and Technology, Gaithersburg, USA).

#### Characterization of soils

The pH was determined in 1:2.5 soil:water suspension after mixing for 12 hours, based on the Hungarian Standard [34]. The organic matter (OM) content of the samples was measured by the modified Walkley-Black method [35], and the calcium carbonate (CaCO_3_) content was measured by the Scheibler gas-volumetric method [34]. The bioavailable forms of P and K was determined using ammonium-acetate lactate extraction (AL-P_2_O_5_; AL-K_2_O) [36], and the total nitrogen content was determined by applying the Kjeldahl method [37]. The ammonium (NH_4_-N) and nitrate (NO_3_-N) concentrations were measured from potassium-chloride (KCl) extract (Sigma Aldrich Ltd., Missouri, USA) based on the Hungarian Standard [38]. The cation exchange capacity (CEC) was determined by using the modified method of Mehlich [39]. The total iodine concentration was quantified by applying an inductively coupled plasma mass spectrometer (ICP-MS), following microwave-assisted aqua regia digestion. The physical and chemical properties of the soils applied are listed in Table 1.

**Table 1:** Physical-chemical properties of soils.

Parameters	Sand	Sandy silt	Silt
pH-H_2_O	7.96	6.83	7.34
Organic matter (w/w%)	0.91	1.24	2.12
CaCO_3_ (w/w%)	1.45	0.08	0.20
Total-N (w/w%)	0.064	0.092	0.135
NH_4_-N (mg kg^-1^)	1.4	2.3	3.9
NO_3_-N (mg kg^-1^)	4.7	2.3	14.2
AL-K_2_O (mg kg^-1^)	74	174	176
AL-P_2_O_5_ (mg kg^-1^)	131	238	81
CEC (Na meq 100 g^-1^)	9	17	37
Total iodine (mg kg^-1^)	1.2	1.9	1.2

#### Plant cultivation and treatment

Plants were grown in a greenhouse (Experimental Station of the Centre of Agricultural Research in Őrbottyán, Hungary), and received natural light. The environmental parameters (temperature, humidity, light intensity) were continuously monitored during the growing period and the measured data has been summarized in Table 2. Seedlings were planted in plastic pots (size: 10 L). At the bottom of all pots, a 4–8 mm thick layer of gravel was placed, which was covered by a synthetic fibrous fabric on which 10 kg of soil was layered. In each pot, three plants were cultivated. For the experiments, three soil types were collected from different regions in Hungary: sand (Mollic Umbrisol (Arenic), Őrbottyán, Hungary), sandy silt (Luvic Calcic Phaeozem, Gödöllő, Hungary), and silt (Calcic Chernozem, Hatvan, Hungary). The plants were irrigated with a mixture of drinking water and Hoagland nutrient solution. During the growing period, the plants (including control) were watered weekly with Hoagland solution. The drinking water used in the experiment was stored in 0.5 m^3^ tanks (separate tanks for each irrigation solution) before application to reduce chlorine concentration. Iodine was added to irrigation water as potassium iodide at concentrations of 0.1 and 0.5 mg I^-^ L^-1^, and an automatic irrigation system delivered the amount of water required for the plants. The water containing trace elements was changed to fresh solutions biweekly in the tanks to eliminate experimental errors caused by changes in concentration. The irrigation and growing parameters of the experiment are listed in Table 3.

**Table 2:** Environmental data of growing period.

Parameter	Pea	Bean
Daytime average temperature (°C)	21.2 ± 7.6	25.5 ± 3.3
Night-time average temperature (°C)	13.7 ± 6.3	18.3 ± 2.3
Photosynthetically active radiation (W/m^2^)	149 ± 91	1045 ± 484
Air humidity (%)	74.0 ± 24.6	70.2 ± 8.6
Soil moisture (% v/v)	23 ± 1	24 ± 3

**Table 3:** Irrigation and growing parameters of bean and pea plants.

	Bean	Pea
Growing period	23 May—24 July	04 April—20 June
Length of growing period (days)	63	78
KI solution (mL)	7750	8680
I load in 0.1 mg/l treatment (mg)	0.775	0.868
I load in 0.5 mg/l treatment (mg)	3.875	4.34

#### In situ measurements of chlorophyll fluorescence (Fv/Fm)

The maximum quantum efficiency of photosystem (PS) II in the youngest adult leaves was typified with an Os30p+ handheld and pulse-modulated fluorometer for in situ determinations of chlorophyll fluorescence (Opti-Sciences, Hudson, USA). The Fv/Fm ratios were calculated to indicate potential stress in the plant caused by iodine (Fv = variable fluorescence level from dark-adapted leaves; Fm = maximal fluorescence level from dark-adapted leaves). The plants were dark-adapted for 30 min, and the Fv/Fm values were determined in all plants immediately before harvest.

#### Sample preparation and elemental analysis of plants

After harvest, plants were cleaned with deionized water, and the root-, aerial-, and edible parts were separated. The aerial part and root samples were dried in a laboratory oven (UF 450 Plus Memmert, Labsystem Ltd., Hungary) at 40°C for two days, while fruits were lyophilized at -70°C in a freeze-dryer Alpha 1 (Christ) equipment (at 200 Pa for 72 hours), and then the dry mass of the different plant parts were determined. Samples were homogenized with a household blender equipped with plastic housing and stainless-steel blades. The dried and homogenized samples were mineralized by applying microwave-assisted acid digestion (TopWave, Analytik Jena, Germany) by adding a mixture of 7 cm^3^ 67% nitric (VWR International, Pennsylvania, USA) and 3 cm^3^ 30% hydrogen-peroxide (VWR International, Pennsylvania, USA) to the dried plant samples (400–500 mg). After digestion, internal standards were added to the digested solution and the volumes were made up to 15 cm^3^ with distilled water. Iodine, macro-, and micronutrient concentrations of the samples were measured by an inductively coupled plasma mass spectrometer (Plasma Quant MS Elite, Analytik Jena, Germany). The NIST Tomato leaf CRM sample recovery values for the investigated eight elements were between 92 and 105%.

### Statistical analysis

Data visualization and statistical analysis were conducted with R statistical software [40]. The line plots used to visualize the mean and standard deviations of data were made with the ggpubr package [41]. Linear models (lm function [40]) were used to compare the effect of different doses in the plants cultivated in different soils. Pair-wise comparisons were carried out with the ‘glht’ function of the ‘multcomp’ package [40,42].

## Results and discussion

### The maximum quantum efficiency of PSII

Chlorophyll fluorescence is one of the most important characteristics of plant photosynthetic performance and viability, indicating the stress status of plants. The maximum quantum efficiency values (Fv/Fm) of photosystem II are listed in Table 4. In both plants, iodine-containing irrigation water caused some changes in the Fv/Fm ratio as compared to the control samples, but the differences were not statistically significant; thus, the results suggest that the treatments did not cause significant stress in the plants. The measured Fv/Fm values ranged between 0.730 to 0.798 and 0.673 to 0.799 in bean and pea plants, respectively. In literature, no experiment has to date focussed on the effect of iodine enrichment on the maximum quantum efficiency of bean plants. Jerše et al. (2018) also did not find a significant effect of iodine spraying treatment (1000 mg L^-1^ KI or 1000 mg L^-1^ KIO_3_) on the morphological, physiological, and biochemical features of the pea plant. The Fv/Fm values ranged from 0.790 to 0.820 in their study, suggesting that the plants were in good condition [20]. These results are similar to our observation, and therefore confirm that the addition of iodine produces no negative effect on the physiological properties of the plants.

**Table 4:** Effect of iodine treatments on the maximum quantum efficiency (Fv/Fm) of photosystem (PS) II in bean and pea plants cultivated in different soils. Data shows the mean of three replicates (RSD%), while different letters show statistically significant differences between treatments (*p* < 0.05; linear regression and Tukey’s test).

Soil type	Iodine concentration of irrigation water	Bean	Pea
Sand	Control	0.735^a^ (2)	0.772^a^ (5)
	0.1 mg L^-1^	0.730^a^ (11)	0.791^a^ (1)
	0.5 mg L^-1^	0.778^a^ (1)	0.777^a^ (2)
Sandy silt	Control	0.764^a^ (4)	0.762^a^ (7)
	0.1 mg L^-1^	0.785^a^ (4)	0.782^a^ (3)
	0.5 mg L^-1^	0.793^a^ (1)	0.673^a^ (22)
Silt	Control	0.773^a^ (3)	0.799^a^ (1)
	0.1 mg L^-1^	0.798^a^ (2)	0.799^a^ (6)
	0.5 mg L^-1^	0.768^a^ (5)	0.724^a^ (6)

### Effect of iodine on the biomass production in bean and pea plants

Dry masses of control and treated plant parts are presented in Table 5. The dry mass of bean roots increased in the order, sandy silt<silt<sandy soil, in all treatment groups, due to the effect of the soil type. Iodine addition produced a negative effect on the biomass production of the bean, however, the changes were significant only in the roots of plants grown in sand soil (*p*<0.01), and in the aerial part (*p*<0.04) and fruits (*p*<0.04) of plants cultivated in silt soil.

**Table 5:** Effect of iodine treatments on the dry mass (g) of different plant parts. Data show the mean of three replicates (RSD%). The different letters show statistically significant differences between the treatments (*p* < 0.05, linear regression, Tukey’s test).

		Bean			Pea		
	Iodine concentration of irrigation water	Root	Aerial part	Fruit	Root	Aerial part	Fruit
Sand	Control	4.82^a^ (11)	16.0^a^ (26)	8.22^a^ (31)	0.80^a^ (36)	4.69^a^ (15)	9.60^a^ (1)
	0.1 mg L^-1^	2.95^b^ (7)	13.1^a^ (39)	9.11^a^ (46)	0.88^a^ (34)	6.26^a^ (16)	9.22^a^ (17)
	0.5 mg L^-1^	2.14^c^ (28)	10.7^a^ (19)	4.86^a^ (32)	0.71^a^ (21)	7.37^b^ (1)	8.34^a^ (30)
Sandy silt	Control	1.63^a^ (9)	12.3^a^ (3)	13.5^a^ (4)	0.60^a^ (27)	6.34^a^ (24)	10.6^a^ (25)
	0.1 mg L^-1^	1.43^a^ (5)	10.0^b^ (13)	13.2^a^ (5)	0.81^a^ (37)	7.14^a^ (32)	10.2^a^ (16)
	0.5 mg L^-1^	1.56^a^ (10)	10.7^a^ (6)	11.1^a^ (13)	0.74^a^ (33)	6.76^a^ (21)	11.0^a^ (4)
Silt	Control	2.01^a^ (9)	13.2^a^ (5)	11.3^a^ (14)	0.65^a^ (32)	5.21^a^ (25)	8.75^a^ (27)
	0.1 mg L^-1^	2.11^a^ (20)	11.7^a^ (16)	10.9^a^ (3)	0.45^a^ (38)	4.20^a^ (16)	10.0^a^ (12)
	0.5 mg L^-1^	2.05^a^ (7)	12.0^a^ (5)	8.04^b^ (14)	0.54^a^ (32)	4.44^a^ (22)	8.97^a^ (13)

In pea, the control plant roots showed a similar but non-significant trend (sandy silt<silt<sand) as compared to bean. However, this trend changed slightly in the treatment groups (i.e., silt<sandy silt<sand for the 0.1 mg I^-^ L^-1^, and silt<sand<sandy silt at the 0.5 mg I^-^ L^-1^ group), but the differences remained non-significant. In the case of roots, moderate changes were observed in the iodine treatment groups as compared to the control samples: a decrement was observed in sandy silt and silt soils, while a 10% increment (0.1 mg I^-^ L^-1^) and 11% reduction (0.5 mg I^-^ L^-1^) were observed in sandy soil, however, the changes were not significant. In the case of the aerial part, the biomass values of the control samples were in the order of sand<silt<sandy silt. In contrast, for treated plants, the lowest values were typical in silt soil, while the highest values were observed in sandy silt soil. At 0.5 mg I^-^ L^-1^, the lowest masses were measured in silt and the highest in the sand soil, however, the changes were significant only in sand soil (*p*<0.08).

In the case of bean fruit, at the iodine concentration of 0.1 mg L^-1^, the biomass increased by 11% only in sandy soil. However, the biomass values decreased by 51%, 18%, and 35% at the iodine dosage of 0.5 mg L^-1^ in sand, sandy silt, and silt soil, respectively. Dobosy et al. (2020a) conducted a rhizo-box experiment with bean plants treated with 0.1–0.5 mg I^-^ L^-1^ [7], in a cultivation medium similar to the present experiment. They observed that iodine addition in concentration of 0.5 mg L^-1^ inhibited the growth of the bean fruit, which is in line with our results.

The biomass of the pea fruit varied in the order of silt<sand<sandy silt at 0.5 mg I^-^ L^-1^, while in the case of 0.1 mg I^-^ L^-1^, the lowest biomass values were observed in sand and the highest in sandy silt soil. The weight of the pea fruits showed a decrement in sand soil, while an increment was observed in sandy silt soil. The silt soil was an exception, with a moderate reduction at 0.1 mg I^-^ L^-1^ and a slight enhancement at 0.5 mg I^-^ L^-1^. Considering the statistical data, the mass variations in the pea fruits were not significant in either case. Jerše et al. (2018), showed that foliar application of 1000 mg L^-1^ KI enhanced the yield of pea fruits (2.58 t/ha) and fresh seed mass (1.22 t/ha), however, the differences as compared to the untreated plants (2.48 t/ha and 1.16 t/ha) and iodide treated plants (2.34 t/ha and 1.12 t/ha) were not statistically significant [20].

### Accumulation and translocation of iodine

**Fig 1:**
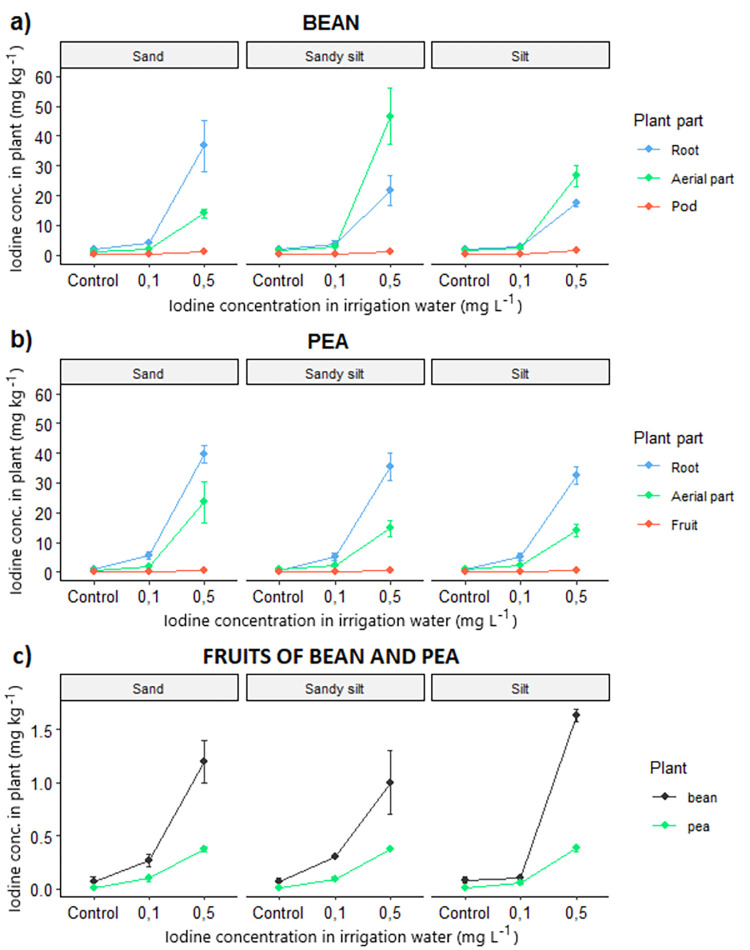
Iodine concentration in the different parts of bean (a) and pea (b) plants, especially focusing on the edible fruits (c). Plants were cultivated in different soils (sand, sandy silt, and silt) and were irrigated with iodine-containing (0.1 and 0.5 mg L^-1^) water.

The average iodine concentration in the different parts of the two plant species grown on different soils is demonstrated in Fig 1.

In the control samples, the iodine concentration of bean and pea fruits varied in the range of 0.05–0.06 and 0.14–0.15 mg kg^-1^, respectively, regardless of the soil type. The iodine concentration in all plant parts increased with higher iodine concentration in the irrigation water in both plants, especially when the dosage was 0.5 mg L^-1^. As a general pattern, the fruit accumulated the least iodine in both species, independent of the soil type. The maximum iodine concentrations (1.6 mg kg^-1^ in bean and 0.4 mg kg^-1^ in pea) were measured when plants were grown in silt soil and irrigated by water containing 0.5 mg I^-^ L^-1^. In the case of bean plant, the root organs accumulated the highest iodine concentration in the sand soil, while in silt soil, the highest values were observed in the aerial parts. In contrast, in the pea plant, the highest iodine concentrations were always detected in the roots. Dobosy et al. (2020a) measured similar maximum iodine concentrations (1.8 mg kg^-1^) in bean fruits in plants grown in the same cultivation medium using 0.5 mg L^-1^ iodine in irrigation water. In a field experiment [20], pea plants were grown in endogenic and gleyic fluvisol soils applying foliar spraying at concentration of 1000 mg I^-^ L^-1^, and it was found that the iodine concentration was more than 6-fold higher in the seeds of the treated plants as compared to the control plants (0.015 mg kg^-1^). In contrast, in our experiment, the highest iodine concentration in the edible part of the treated plant (0.37–0.38 mg kg^-1^) was only 3-fold higher as compared to the control plant (0.14–0.15 mg kg^-1^), but the two experiments are not comparable due to different experimental set-up and iodine concentrations applied.

Foliar spray treatments with KI and KIO_3_ (5, 10 and 15 kg I^-^ ha^-1^) were used in cowpea plants, and it was concluded that iodide treatment seemed to be more effective than iodate and maximum ioidne concetration was amounted to 5854 mg/kg by applying 15 kg I^-^ ha^-1^ dosage [32].

The distribution of iodine among the plant parts is listed in Table 6, and the values were calculated based on the dry mass and the iodine concentration. The highest iodine accumulation (bean: 59–92% and pea: 64–90%) was observed in the aerial parts, and only 2–11% and 2–6% were translocated to the bean and pea fruits, respectively. In our former experiment, uptake and translocation of iodine was studied in bean plants grown on calcareous sandy soil and irrigated with water containing 0.10, 0.25, and 0.50 mg I^-^ L^-1^ [7]. We observed a different distribution pattern (i.e., only 1.0% of iodine accumulated in the fruits, while 83–87% accumulated in the roots), which can be explained by the different scales of the experiments. Iodine in plants is transported mainly through the xylem, therefore, the accumulation of iodine in the edible parts is less efficient [25,23].

**Table 6:** Iodine distribution (as % of g total uptake) among the bean and pea plant parts.

		Bean			Pea		
	Iodine concentration of irrigation water	Root	Aerial part	Fruit	Root	Aerial part	Fruit
Sand	Control	39	59	2	22	74	4
	0.1 mg L^-1^	30	63	7	30	64	6
	0.5 mg L^-1^	34	64	2	14	84	2
Sandy silt	Control	13	84	3	7	90	3
	0.1 mg L^-1^	14	75	11	20	75	5
	0.5 mg L^-1^	6	92	2	20	77	3
Silt	Control	17	80	3	13	85	2
	0.1 mg L^-1^	13	84	3	18	77	5
	0.5 mg L^-1^	10	87	3	21	75	4

### Effect of iodine on the transport of selected macro-and microelements

The concentration of selected macro-and microelements in the different plant parts of bean and pea plants are listed in Table 7. In the fruit of the bean plant, the concentrations of Mg, P, Mn, Fe, and Zn were stimulated by the iodine treatments (*p*<0.03), especially when the iodine dosage was 0.5 mg I^-^ L^-1^, while the concentrations of Cu and B did not change as compared to the control samples. In the case of pea fruit, iodine treatment resulted in a decrement in Zn concentration (*p*<0.02) but stimulated (*p*<0.04) the Fe concentration. The concentration of Mg and P showed an increment at 0.1 mg I^-^ L^-1^, and a significant reduction (*p*<0.0.4) at a dose of 0.5 mg I^-^ L^-1^, while the concentrations of Mn, Cu, and B did not show any changes as compared to the control samples. In literature, a limited number of studies have examined the effect of iodine supplementation on the concentration of different nutrients. Based on our former studies, the presence of iodine can significantly influence the concentrations of macro-and microelements in cabbage, tomato, carrot, and potato plants, especially at 0.5 mg I^-^ L^-1^ [7–9]. Dobosy et al. (2020a) found that the concentration changes of essential elements were negligible in bean fruits [7], but in our experiment, the concentration of nutrients increased in some cases at 0.5 mg I^-^ L^-1^ in all soils, and this increment was also statistically significant (*p*<0.03) in the case of Mg, P, Mn, Fe, and Zn. These results suggest that monitoring the impact of iodine enrichment on micro-and macro elements concentration should be emphasized in the future.

**Table 7:** Average macro-and micronutrient concentrations of bean and pea plant parts cultivated in three different soils and irrigated with water containing iodine. Data show the means of three replicates (RSD%) and the different letters show statistically significant differences between treatments (*p* < 0.05; linear regression and Tukey’s test).

Bean										
		Sand			Sandy silt			Silt		
		Root	Aerial part	Fruit	Root	Aerial part	Fruit	Root	Aerial part	Fruit
Mg (mg kg^-1^)	Control	8074 ± 184^a^	4431 ± 18^a^	2342 ± 81^a^	4699 ± 325^a^	4528 ± 51^a^	2573 ± 175^a^	5845 ± 277^a^	6914 ± 21^a^	2876 ± 199^a^
	0.1 mg L^-1^	8087 ± 140^a^	5095 ± 342^a^	2873 ± 495^a^	4049 ± 830^b^	5216 ± 189^b^	3063 ± 115^a^	5693 ± 556^a^	7211 ± 425^a^	3004 ± 152^a^
	0.5 mg L^-1^	14000 ± 463^b^	5357 ± 381^b^	3091 ± 414^a^	4640 ± 537^a^	4644 ± 212^c^	3136 ± 270^b^	8259 ± 137^b^	7123 ± 375^a^	3896 ± 398^b^
P (mg kg^-1^)	Control	4823 ± 1262^a^	2856 ± 512^a^	3641 ± 33^a^	3740 ± 228^a^	2974 ± 163^a^	3845 ±233^a^	4232 ± 384^a^	3385 ± 319^a^	4502 ± 241^a^
	0.1 mg L^-1^	4806 ± 527^a^	2685 ± 344^a^	4731 ± 1250^b^	3829 ± 239^a^	3219 ± 129^a^	4658 ± 194^b^	4319 ± 196^a^	3358 ± 234^a^	4726 ± 190^a^
	0.5 mg L^-1^	4998 ± 227^a^	4308 ± 233^b^	5887 ± 482^c^	4387 ± 105^b^	3606 ± 177^b^	5001 ± 609^c^	5226 ± 269^b^	3967 ± 169^a^	6210 ± 897^b^
Mn (mg kg^-1^)	Control	84 ± 17^a^	69 ± 21^a^	24 ± 2^a^	24 ± 3^a^	65 ± 12^a^	21 ± 1^a^	32 ± 11^a^	69 ± 7^a^	18 ±2^a^
	0.1 mg L^-1^	121 ± 78^a^	98 ± 9^a^	32 ± 14^a^	25 ± 2^a^	83 ± 10^a^	24 ± 2^a^	26 ± 3^a^	70 ± 5^a^	18 ± 1^a^
	0.5 mg L^-1^	102 ± 20^a^	104 ± 26^a^	38 ± 11^a^	37 ± 4^b^	85 ± 2^a^	29 ± 3^b^	58 ± 38^b^	76 ± 10^a^	27 ± 6^a^
Fe (mg kg^-1^)	Control	1415 ± 628^a^	152 ± 8^a^	99 ± 8^a^	920 ± 99^a^	221 ± 38^a^	76 ± 3^a^	1007 ± 356^a^	237 ± 10^a^	76 ± 4^a^
	0.1 mg L^-1^	1314 ± 553^a^	192 ± 2^a^	117 ± 10^a^	911 ± 84^a^	202 ± 10^a^	99 ± 32^a^	840 ± 200^a^	214 ± 36^a^	79 ± 4^a^
	0.5 mg L^-1^	1205 ± 350^a^	237 ± 43^b^	131 ± 29^a^	1464 ± 222^b^	238 ± 14^a^	117 ± 7^a^	1444 ± 838^a^	252 ± 60^a^	138 ± 26^b^
Cu (mg kg^-1^)	Control	8 ± 2^a^	95 ± 43^a^	4 ± 0^a^	6 ± 2^a^	164 ± 32^a^	4 ± 0^a^	6 ± 3^a^	204 ± 28^a^	4 ± 1^a^
	0.1 mg L^-1^	4 ± 1^a^	103 ± 42^a^	4 ± 1^a^	5 ± 1^a^	225 ± 5^b^	6 ± 0^a^	6 ± 2^a^	191 ± 17^a^	4 ± 0^a^
	0.5 mg L^-1^	5 ± 0^a^	57 ± 19^a^	5 ± 1^a^	8 ± 4^a^	182 ± 12^a^	6 ± 1^a^	8 ± 2^a^	170 ± 14^a^	6 ± 1^b^
Zn (mg kg^-1^)	Control	206 ± 42^a^	48 ± 4^a^	45 ± 2^a^	236 ± 138^a^	51 ± 2^a^	47 ± 2^a^	176 ± 10^a^	49 ± 2^a^	48 ± 3^a^
	0.1 mg L^-1^	189 ± 32^a^	42 ± 4^a^	49 ± 5^a^	173 ± 22^a^	55 ± 1^a^	56 ± 5^a^	186 ± 28^a^	49 ± 7^a^	52 ± 7^a^
	0.5 mg L^-1^	181 ± 3^a^	48 ± 1^a^	51 ± 5^a^	188 ± 12^a^	57 ± 13^a^	58 ± 8^a^	209 ± 33^a^	46 ± 1^a^	67 ± 8^b^
B (mg kg^-1^)	Control	21 ± 2^a^	24 ± 2^a^	20 ± 4^a^	18 ± 1^a^	27 ± 3^a^	16 ± 1^a^	19 ± 3^a^	28 ± 3^a^	18 ± 2^a^
	0.1 mg L^-1^	22 ± 5^a^	29 ± 1^a^	19 ± 1^a^	16 ± 2^a^	33 ± 1^a^	17 ± 1^a^	17 ± 2^a^	31 ± 3^a^	15 ± 1^a^
	0.5 mg L^-1^	19 ± 2^a^	53 ± 11^b^	29 ± 10^a^	14 ± 1^a^	40 ± 1^a^	23 ± 2^a^	19 ± 1^a^	38 ± 2^a^	21 ± 3^a^
Pea										
		Sand			Sandy silt			Silt		
		Root	Aerial part	Fruit	Root	Aerial part	Fruit	Root	Aerial part	Fruit
Mg (mg kg^-1^)	Control	8843 ± 516^a^	5611 ± 673^a^	1620 ± 201^a^	3352 ± 418^a^	4242 ± 630^a^	1502 ± 181^a^	4851 ± 547^a^	4492 ± 829^a^	1713 ± 199^a^
	0.1 mg L^-1^	12081 ± 1199^b^	5922 ± 590^a^	1790 ± 258^a^	6065 ± 2273^a^	4619 ± 886^a^	1760 ± 179^a^	6542 ± 1246^a^	5866 ± 1006^a^	1976 ± 54^a^
	0.5 mg L^-1^	11447 ± 888^b^	5148 ± 349^a^	1263 ± 130^b^	4555 ± 718^a^	5155 ± 52^a^	1288 ± 75^b^	6355 ± 975^a^	5300 ± 333^a^	1440 ± 135^b^
P (mg kg^-1^)	Control	5668 ± 223^a^	2559 ± 140^a^	7460 ± 935^a^	6838 ± 1101^a^	2728 ± 353^a^	7873 ± 659^a^	7326 ± 1385^a^	2023 ± 241^a^	7585 ± 121^a^
	0.1 mg L^-1^	8369 ± 512^b^	3409 ± 368^b^	10052 ± 520^b^	8016 ± 476^a^	2819 ± 158^a^	10003 ± 1229^b^	7341 ± 300^a^	2012 ± 275^a^	10318 ± 178^b^
	0.5 mg L^-1^	10552 ± 326^c^	3757 ± 116^b^	6210 ± 734^a^	9867 ± 502^b^	3764 ± 173^b^	7126 ± 195^a^	10200 ± 1241^b^	2061 ± 296^a^	7103 ± 820^a^
Mn (mg kg^-1^)	Control	62 ± 3^a^	84 ± 4^a^	16 ± 1^a^	31 ± 2^a^	51 ± 4^a^	12 ± 1^a^	63 ± 5^a^	70 ± 3^a^	16 ± 1^a^
	0.1 mg L^-1^	62 ± 2^a^	78 ± 10^a^	15 ± 2^a^	27 ± 3^a^	50 ± 7^a^	12 ± 0^a^	60 ± 3^a^	95 ± 3^b^	17 ± 1^a^
	0.5 mg L^-1^	114 ± 18^b^	76 ± 8^a^	15 ± 1^a^	52 ± 6^b^	60 ± 5^a^	12 ± 1^a^	103 ± 9^b^	116 ± 10^c^	17 ± 1^a^
Fe (mg kg^-1^)	Control	1249 ± 236^a^	152 ± 12^a^	61 ± 5^a^	619 ± 69^a^	152 ± 20^a^	77 ± 1^a^	1393 ± 163^a^	217 ± 4^a^	78 ± 11^a^
	0.1 mg L^-1^	1970 ± 125^b^	177 ± 33^a^	103 ± 24^b^	1075 ± 152^a^	247 ± 40^b^	112 ± 4^b^	1358 ± 140^a^	312 ± 37^b^	92 ± 2^a^
	0.5 mg L^-1^	2509 ± 400^c^	163 ± 19^a^	62 ± 1^a^	1494 ± 304^b^	203 ± 15^a^	91 ± 6^c^	2472 ± 26^b^	246 ± 27^a^	80 ± 5^a^
Cu (mg kg^-1^)	Control	19 ± 2^a^	4 ± 0^a^	7 ± 1^a^	21 ± 8^a^	4 ± 1^a^	8 ± 1^a^	22 ± 2^a^	5 ± 1^a^	8 ± 1^a^
	0.1 mg L^-1^	24 ± 2^a^	4 ± 0^a^	7 ± 1^a^	25 ± 4^a^	5 ± 0^a^	7 ± 0^a^	23 ± 1^a^	4 ± 1^a^	7 ± 0^a^
	0.5 mg L^-1^	21 ± 2^a^	4 ± 1^a^	6 ± 0^a^	24 ± 4^a^	4 ± 0^a^	8 ± 1^a^	29 ± 2^b^	5 ± 1^a^	7 ± 0^a^
Zn (mg kg^-1^)	Control	245 ± 23^a^	48 ± 4^a^	71 ± 5^a^	287 ± 19^a^	102 ± 7^a^	87 ± 2^a^	330 ± 23^a^	41 ±2^a^	87 ± 4^a^
	0.1 mg L^-1^	227 ± 21^a^	33 ± 3^b^	54 ± 7^b^	225 ± 17^b^	70 ± 2^b^	77 ± 5^a^	302 ± 34^b^	34 ± 5^a^	63 ±7^b^
	0.5 mg L^-1^	178 ± 25^b^	26 ± 1^c^	47 ± 3^c^	178 ± 8^c^	62 ± 6^c^	76 ± 7^a^	203 ± 28^c^	46 ± 3^b^	64 ± 5^b^
B (mg kg^-1^)	Control	17 ± 1^a^	30 ± 3^a^	12 ± 1^a^	18 ± 0^a^	52 ± 7^a^	10 ± 1^a^	18 ± 2^a^	35 ± 1^a^	14 ± 5^a^
	0.1 mg L^-1^	18 ± 3^a^	23 ± 3^a^	10 ± 1^a^	21 ± 7^a^	36 ± 5^b^	11 ± 1^a^	17 ± 2^a^	30 ± 4^a^	11 ± 1^a^
	0.5 mg L^-1^	17 ± 2^a^	33 ± 7^a^	11 ± 1^a^	19 ± 2^a^	43 ± 6^a^	10 ± 1^a^	19 ± 1^a^	32 ± 3^a^	11 ± 1^a^

## Conclusions

In this study, the biofortification of green bean and garden pea with iodine and the effect of iodine on the maximum quantum efficiency, biomass production, and selected essential macro- and microelement concentrations of plants cultivated in sand, sandy silt, and silt soils have been discussed. On the basis of our experimental results, it can be concluded that the green bean would be a promising target plant for biofortification with iodine. However, it should be noted that the increase in iodine concentration of bean fruits and additionally, the increase in concentrations of Mg, P, Fe, and Mn is accompanied by a decrease in biomass production. The extent of this phenomenon is strongly dependent on the soil type, with an increasing tendency of biomass reduction in the following order: sandy silt< silt<sand.

In our further experiments the valence state of iodine will be studied by X-ray absorption near-edge spectroscopy technique to clarify the chemical form of the accumulated iodine in the plants.

## Supporting information

S1 File (XLSX)

S1 Graphical abstract (JPEG)
