# Supplementary figures and images for: Iodine biofortification of bean (Phaseolus vulgaris L.) and pea (Pisum sativum L.) plants cultivated in three different soils
Source: PLoS One. 2022 Oct 4;17(10):e0275589. doi: 10.1371/journal.pone.0275589 (PMC9531830; doi:10.1371/journal.pone.0275589)

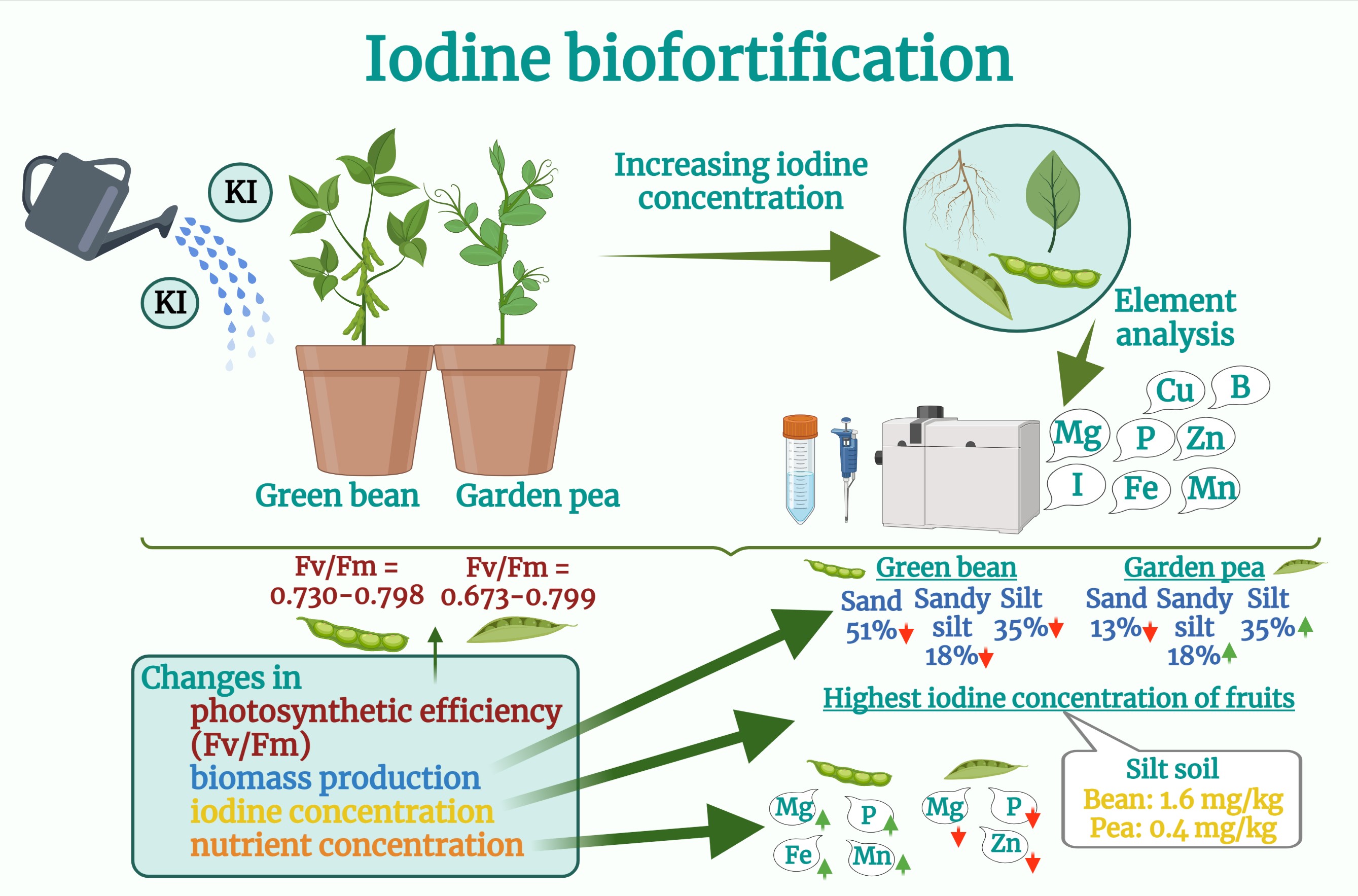

Supplement: S1 Graphical abstract — (JPEG) [file pone.0275589.s002.jpeg]
